# Draft Genome Sequences of Two Enterobacter hormaechei subsp. *xiangfangensis* Strains Isolated from the Moroccan Sahara

**DOI:** 10.1128/mra.01087-22

**Published:** 2023-01-12

**Authors:** Houda Zouagui, Amina Manni, Meriem Laamarti, Souad Kartti, Tarek Alouane, Lamiaa Lahlou, Oussama Benhrif, Loubna Temsamani, Jamal-Eddine Eljamali, Abdelkarim Filali-Maltouf, Azeddine Ibrahimi, Laila Sbabou

**Affiliations:** a Laboratory of Microbiology and Molecular Biology, Faculty of Sciences, Mohammed V University in Rabat, Rabat, Morocco; b Biotechnology Laboratory, Bioinova Research Center, Rabat Medical and Pharmacy School, Mohammed V University in Rabat, Rabat, Morocco; c Mohammed VI University of Health Sciences, Casablanca, Morocco; Wellesley College

## Abstract

We report the draft genome sequences of Enterobacter hormaechei subsp. *xiangfangensis* strains MDMC82 and MDMC76, which were isolated from the sand dunes of the Merzouga desert in the Moroccan Sahara. These bacteria are able to tolerate the harsh environmental conditions of the Moroccan desert.

## ANNOUNCEMENT

Deserts are one of the Earth’s important ecosystems; they are characterized by desiccation, intense solar radiation, and lack of nutrients (1). Merzouga is a region in southeast Morocco that is known for its desert climate. It represents an arid desert ecosystem in which any living organism requires specific abilities to survive these challenging circumstances.

In this study, we report the draft genome sequences of two Enterobacter hormaechei subsp. *xiangfangensis* strains (MDMC82 and MDMC76), which were isolated from nonvegetated sand dunes. The samples were collected at a depth of 0 to 5 cm in the Merzouga region (31°6′33.6″N, 3°58′42.675″W).

The isolates were obtained from 1-g sand samples, which were suspended in 9 mL of physiological water and shaken vigorously for 30 min. From this stock, serial dilutions (10^−3^ to 10^−7^) were prepared. A 0.1-mL aliquot from each dilution was plated on nutrient agar supplemented with 50 μg/mL amphotericin B and was incubated at 28°C for 2 days. The strains were subsequently purified and stored in 40% glycerol solution at −80°C.

Genomic DNA was extracted from a liquid nutrient broth following the phenol-chloroform method (2). The DNA samples were quantified using a Quantus fluorometer (Promega) and run on a 1% agarose gel. The sequencing library was prepared using the Nextera XT DNA sample preparation kit (Illumina, Inc., San Diego, CA, USA). Paired-end 300-bp reads were generated on the MiSeq platform using the MiSeq reagent kit v3 (600 cycles; Illumina).

The quality of the sequencing data was assessed using FastQC v0.11.6 (3). Trimming and removal of low-quality sequences were performed using Trimmomatic v0.39 (4). *De novo* assembly was performed with SPAdes v3.11.1 (5) and assessed with QUAST v5.0.0 (6). The total sequence lengths are 4,796,963 bp (MDMC82) and 4,786,737 bp (MDMC76). Further genomic data are summarized in Table 1.

**TABLE 1:** Genomic features of the strains

Feature^*a*^	Finding for:	
	MDMC82	MDMC76
No. of reads	1,087,196	1,671,424
No. of contigs	267	236
Genome coverage (×)	57	75
Contig *N*_50_ (bp)	226,473	150,767
Total sequence length (bp)	4,796,963	4,786,737
GC content (%)	55.2	55.1
No. of CDSs	4,661	4,631
No. of rRNAs	19	33
No. of tRNAs	79	79
No. of ncRNAs	8	6
No. of pseudogenes	156	156
ANIm best match (%)	99.12	99.12
dDDH best match (%)	92.4	92.2

a CDS, coding DNA sequence; ncRNA, noncoding RNA.

Annotation was performed using the RAST Server v2.0 (7) and the NCBI Prokaryotic Genome Annotation Pipeline (PGAP) v5.3 (8). Strain identification was carried out using the Type (Strain) Genome Server (TYGS) (9). Multilocus sequence typing (MLST) was performed using the Enterobacter cloacae typing scheme on PubMLST (10). The identification was confirmed by average nucleotide identity calculated using MUMmer average nucleotide identity (ANIm) calculations with Pyani v0.2.9 (11) and digital DNA-DNA hybridization (dDDH) calculations using formula 2 with the Genome-to-Genome Distance Calculator (GGDC) v2.1 (https://ggdc.dsmz.de/ggdc.php) (12). Enterobacter hormaechei subsp. *xiangfangensis* type strain LMG27195 (GenBank accession number CP017183.1) showed the best match ANIm and dDDH values for the two isolates (Table 1). Default parameters were used for all software tools.

### Data availability.

The genome assemblies were deposited in DDBJ/ENA/GenBank under the accession numbers SWCT01000000 (MDMC82) and SWCU01000000 (MDMC76), with the BioSample accession numbers SAMN11483247 (MDMC82) and SAMN11483246 (MDMC76). The raw reads were deposited in the NCBI Sequence Read Archive (SRA) under the BioProject accession number PRJNA886995, with the SRA accession numbers SRX17793474 and SRX17793473 for MDMC82 and MDMC76, respectively.

## References

[1] Perera I, Subashchandrabose SR, Venkateswarlu K, Naidu R, Megharaj M. 2018. Consortia of cyanobacteria/microalgae and bacteria in desert soils: an underexplored microbiota. Appl Microbiol Biotechnol 102:7351–7363. doi:10.1007/s00253-018-9192-1. 29982925

[2] Chen WP, Kuo TT. 1993. A simple and rapid method for the preparation of Gram-negative bacterial genomic DNA. Nucleic Acids Res 21:2260. doi:10.1093/nar/21.9.2260. 8502576 PMC309503

[3] Andrews S. 2010. FastQC: a quality control tool for high throughput sequence data. http://www.bioinformatics.babraham.ac.uk/projects/fastqc.

[4] Bolger AM, Lohse M, Usadel B. 2014. Trimmomatic: a flexible trimmer for Illumina sequence data. Bioinformatics 30:2114–2120. doi:10.1093/bioinformatics/btu170. 24695404 PMC4103590

[5] Bankevich A, Nurk S, Antipov D, Gurevich AA, Dvorkin M, Kulikov AS, Lesin VM, Nikolenko SI, Pham S, Prjibelski AD, Pyshkin AV, Sirotkin AV, Vyahhi N, Tesler G, Alekseyev MA, Pevzner PA. 2012. SPAdes: a new genome assembly algorithm and its applications to single-cell sequencing. J Comput Biol 19:455–477. doi:10.1089/cmb.2012.0021. 22506599 PMC3342519

[6] Gurevich A, Saveliev V, Vyahhi N, Tesler G. 2013. QUAST: quality assessment tool for genome assemblies. Bioinformatics 29:1072–1075. doi:10.1093/bioinformatics/btt086. 23422339 PMC3624806

[7] Aziz RK, Bartels D, Best AA, DeJongh M, Disz T, Edwards RA, Formsma K, Gerdes S, Glass EM, Kubal M, Meyer F, Olsen GJ, Olson R, Osterman AL, Overbeek RA, McNeil LK, Paarmann D, Paczian T, Parrello B, Pusch GD, Reich C, Stevens R, Vassieva O, Vonstein V, Wilke A, Zagnitko O. 2008. The RAST Server: Rapid Annotations using Subsystems Technology. BMC Genomics 9:75. doi:10.1186/1471-2164-9-75. 18261238 PMC2265698

[8] Tatusova T, DiCuccio M, Badretdin A, Chetvernin V, Nawrocki EP, Zaslavsky L, Lomsadze A, Pruitt KD, Borodovsky M, Ostell J. 2016. NCBI Prokaryotic Genome Annotation Pipeline. Nucleic Acids Res 44:6614–6624. doi:10.1093/nar/gkw569. 27342282 PMC5001611

[9] Meier-Kolthoff JP, Göker M. 2019. TYGS is an automated high-throughput platform for state-of-the-art genome-based taxonomy. Nat Commun 10:2182. doi:10.1038/s41467-019-10210-3. 31097708 PMC6522516

[10] Jolley KA, Bray JE, Maiden MCJ. 2018. Open-access bacterial population genomics: BIGSdb software, the PubMLST.org website and their applications. Wellcome Open Res 3:124. doi:10.12688/wellcomeopenres.14826.1. 30345391 PMC6192448

[11] Pritchard L, Glover R, Humphris S, Elphinstone J, Toth I. 2016. Genomics and taxonomy in diagnostics for food security: soft-rotting enterobacterial plant pathogens. Anal Methods 8:12–24. doi:10.1039/C5AY02550H.

[12] Meier-Kolthoff JP, Auch AF, Klenk H-P, Göker M. 2013. Genome sequence-based species delimitation with confidence intervals and improved distance functions. BMC Bioinformatics 14:60. doi:10.1186/1471-2105-14-60. 23432962 PMC3665452

